# Using High Angular Resolution Diffusion Imaging Data to Discriminate Cortical Regions

**DOI:** 10.1371/journal.pone.0063842

**Published:** 2013-05-17

**Authors:** Zoltan Nagy, Daniel C. Alexander, David L. Thomas, Nikolaus Weiskopf, Martin I. Sereno

**Affiliations:** 1 Wellcome Trust Centre for Neuroimaging, UCL Institute of Neurology, University College London, London, United Kingdom; 2 Centre for Medical Image Computing, Department of Computer Science, University College London, London, United Kingdom; 3 Department of Brain Repair and Rehabilitation, UCL Institute of Neurology, University College London, London, United Kingdom; 4 Cognitive, Perceptual and Brain Sciences, University College London, London, United Kingdom; 5 Department of Psychology, Birkbeck College, University of London, United Kingdom; University of Minnesota, United States of America

## Abstract

Brodmann’s 100–year–old summary map has been widely used for cortical localization in neuroscience. There is a pressing need to update this map using non–invasive, high–resolution and reproducible data, in a way that captures individual variability. We demonstrate here that standard HARDI data has sufficiently diverse directional variation among grey matter regions to inform parcellation into distinct functional regions, and that this variation is reproducible across scans. This characterization of the signal variation as non–random and reproducible is the critical condition for successful cortical parcellation using HARDI data. This paper is a first step towards an individual cortex–wide map of grey matter microstructure, The gray/white matter and pial boundaries were identified on the high–resolution structural MRI images. Two HARDI data sets were collected from each individual and aligned with the corresponding structural image. At each vertex point on the surface tessellation, the diffusion–weighted signal was extracted from each image in the HARDI data set at a point, half way between gray/white matter and pial boundaries. We then derived several features of the HARDI profile with respect to the local cortical normal direction, as well as several fully orientationally invariant features. These features were taken as a fingerprint of the underlying grey matter tissue, and used to distinguish separate cortical areas. A support–vector machine classifier, trained on three distinct areas in repeat 1 achieved 80–82% correct classification of the same three areas in the unseen data from repeat 2 in three volunteers. Though gray matter anisotropy has been mostly overlooked hitherto, this approach may eventually form the foundation of a new cortical parcellation method in living humans. Our approach allows for further studies on the consistency of HARDI based parcellation across subjects and comparison with independent microstructural measures such as ex–vivo histology.

## Introduction

A century after the publication of Brodmann's classic cytoarchitectonic maps of the cortex in humans and other species [1], [2], cortical parcellation remains a difficult unsolved problem. Workers following Brodmann distinguished additional areas beyond the approximately 50 areas he labeled using both cytoarchitecture [3] and especially myeloarchitecture. Vogt and Vogt, for example, recognized almost 200 myeloarchitectonic areas in each hemisphere [4]. After an additional century of research, unsurprisingly, some flaws were uncovered. For example, Brodmann did not identify primate area MT/V5, an area now known to characterize in all primates. Also, those maps did not quantify regional features nor between–subject variability, and relationships with function were just beginning to be made. Yet Brodmann’s first human map is still widely used in human functional neuroimaging studies. This is partly because the enormous amount of manual work that was required to construct that map has only recently been surpassed. Finally, human structural neuroimaging methods have so far not been able to provide a rich enough feature set to distinguish most cortical areas.

The human neocortex is 2–4 mm thick and is conventionally divided into 6 main layers [5]. Following Meynert’s recognition in 1867 of regional (macroscopically visible) anatomical differences in the cortex [6], it has become accepted that histological variability defines cortical areas [7] in which the constituent layers can vary in cell type, neurotransmitter receptor type, extent of myelination and thickness. These specializations reflect both intrinsic connections within an area and extrinsic connections of that area with the rest of the brain and are thought to form the anatomical basis of functional localization [8]–[11]. Such anatomical specialization may also indicate the subtle differences between the computations performed in different cortical areas. Cortical thickness development has a dynamic nature during childhood and early adulthood [12], [13]. Cortical thickness has also been found to correlate with functional ability [14], disability [15], age [16] and be affected by pre– and perinatal events [17], [18].

More recently, much effort has been aimed at using magnetic resonance imaging (MRI) to quantitatively map out the histology of the human cortex at a uniformly fine spatial resolution, non–invasively and at an individual level. However, the choice of appropriate MRI contrast is not obvious [19], [20].

Diffusion tensor imaging (DTI), which measures the diffusion constant in three dimensions [21], has primarily been used to scrutinize the microstructure of brain white matter (WM) where the diffusivity is highly anisotropic (directionally dependent), reflecting local axonal fiber orientation [22]. Although analysis of voxels containing cortical gray matter (GM) results in less pronounced anisotropy, some investigators have found it possible to follow developmental changes of cortical GM in fetuses [23] or to parcellate the subcortical GM nuclei of the amygdala [24]. At 7T [25] and in ex–vivo preparations [26], [27], previous investigators have also identified anisotropic diffusion profiles in cortical areas of adult humans.

High angular resolution diffusion imaging (HARDI) acquisitions [28], [29] collect diffusion–weighted images with a fixed b–value and many more gradient directions than the minimum six required for DTI. A HARDI acquisition ensures stability and reliability of the diffusion tensor estimate [30] and supports complex models of fibre orientation distribution in voxels where the diffusion tensor model is not appropriate – e.g., at the boundaries between different tissue types or crossing WM fiber pathways (see [31], [32] for review). One way to represent the HARDI signal is via the spherical harmonic series and early work on HARDI data [33], [34] shows significant departures of the signal from the diffusion tensor model in WM and especially at known fibre crossings. Since those early demonstrations, researchers have devised a wide range of models and algorithms to exploit the HARDI signal for estimating increasingly subtle features of WM tissue such as fibre orientation distribution [31], [32], axon diameter and density [35], or both [36].

The HARDI signal in GM has received less attention, although a few studies reveal that it does provide useful contrast in GM voxels. Deoni et al. [37] used a “time–series”–like representation of the HARDI signals to parcellate the nuclei of the thalamus. Rather than fitting a model to the data to describe the local diffusion profile they used the pattern of signal amplitudes directly. High correlation of the series of signals was taken to identify homologous tissue types. However, their method is not rotationally invariant and thus the HARDI signal profile would produce low correlation even from identical tissue if its orientation was varied. Thus the method is difficult to extend directly to the cortex, where the dominant orientation varies widely as a result of cortical folding. To this end Haroon et al. [38] use the histogram of peak counts in the Q–ball ODF [39] over multiple bootstrap experiments as a feature of local GM architecture. This feature is independent of orientation and so is able to associate voxels with similar microstructure but different orientation.

Here we introduce a set of features, estimated directly from the HARDI signal, that capture the intrinsic 3D shape of the diffusion profile and relate it to the local surface normal. We demonstrate that these features contain sufficient information to discriminate functionally distinct areas of the cortex in live human volunteers scanned using a clinical MRI system. The findings show that the diffusion signal originating from GM, although much more spatially isotropic than that from WM, has sufficient and reliable signal variation to characterize and discriminate the tissue. This work is a step toward the ultimate goals of identifying cortical boundaries based on variation in tissue microstructure, initially recognized in post–mortem specimens by Brodmann and others [1], [3], [4], [11] and parcellating the entire cortical area in an unsupervised fashion.

## Methods

### Ethics Statement

Ethics approval for this study was obtained from the National Hospital for Neurology and Neurosurgery and Institute of Neurology Joint Research Ethics Committee. The three adult subjects (2 male) gave informed written consent to participation in accordance with the approval of this ethics committee.

### MRI Data Acquisition

HARDI data were collected on a 3T scanner (Tim Trio, Siemens Healthcare, Erlangen, Germany) with a radio frequency body transmit and 32–channel receive–only head coil. The subjects’ head was carefully immobilized within the tight geometry of the head coil. Two datasets were collected with a custom–made sequence [40] at 2.3 mm^3^ isotropic resolution in order to measure test/re–test variability. The two data sets also provided independent training and test sets for classification experiments (see below). Each dataset consisted of one reference image with b = 100 s/mm^2^ and 61 diffusion weighted images (DWIs) with b = 1000 s/mm^2^ and diffusion directions distributed evenly on the surface of a sphere [41]. In a separate session structural images were collected at 0.8 mm isotropic resolution with two types of contrast weightings (proton density and T1). After correction for transmit inhomogeneity using a measured B_1_^+^ map [42], [43] a quantitative T1–map was calculated [44]. The T1 map was used for the accurate identification of the GM/WM boundary and the pial surface for the purposes of sampling the HARDI data as well as for displaying results. Table 1 shows the detailed acquisition parameters of all imaging protocols.

**Table 1:** MRI data acquisition parameters.

Sequence	Resolution (mm^3^)	TE (ms)	TR (ms)	Matrix	# of Slices/Partitions	Flip Angle
HARDI	2.3×2.3×2.3	90	7300	96×96	52	90°
B1 Map	4×4×4	39, 73	500	64×48	48	*
FLASH	0.8×0.8×0.8	2.2, 4.7, 7.3, 9.8	23.7	320×270	240	6° or 28°

* B1 Map uses two echoes, one spin echo and one stimulated echo. The flip angles were varied between 270°–130° in steps of 10° for the spin echo and between 135°–65° in steps of 5° for the stimulated echo [43].

The T1 map has considerably higher resolution than the DWIs. This is crucial for accurately identifying the GM/WM and pial boundaries and reduces noise in the estimation of the direction of the local vector normal to the GM/WM boundary. Both of these aspects improve the precision of sampling the lower resolution DWI signal from the HARDI data set (see below).

### Preparation of DWIs for Cortical Signal Intensity Extraction

The GM/WM and pial surfaces were identified in FreeSurfer [45], [46]. The test/re–test DWIs were first aligned to each other on a per diffusion encoding direction basis in AFNI (http://afni.nimh.nih.gov/afni) (using 3dvolreg, heptic interpolation) to correct for discrepancies between the datasets. Pooled across the subjects, the maximum ranges of the amount of movement correction between the two datasets were well under a degree for rotation and under a millimeter for translation, except along the phase–encoding direction where apparent motion, due to B_o_ drift, was observed. Since, the professional volunteers were highly compliant, as demonstrated by the minimal amount of correction required across data sets, the only additional within–data–set correction applied was the monotonic image translation to cancel the B_0_ drift along the phase encoding direction. Specifically, the measured translation, T, between successive reference (b = 100 s/mm^2^) image volumes at the beginning of the test and retest datasets was used move the above aligned test–retest DWIs incrementally (i.e. 1*T/68 for the first image volume, 2*T/68 for the second image volume, etc).

The first reference DWI image was then aligned with quantitative T1 map images using manual blink comparison (contrast–inverting in–house version of FreeSurfer tkregister). The resulting 4×4, affine, transformation matrix was used to align the 61 directions of B_o_ drift–corrected HARDI data with the higher resolution GM/WM surface reconstruction (approximately 150,000 vertices per hemisphere). From each vertex point on the GM/WM boundary surface, the direction of the local normal vector was followed to half way between the GM/WM boundary and the pial surface. At that point a single sample of signal intensity was taken from each, aligned DWI. The diffusion data for each direction was smoothly interpolated (within–direction) onto the higher resolution surface mesh using iterative nearest neighbor estimation [47]. The estimated FWHM was a 1.8 mm surface kernel, which was smaller than the resolution of the DWI images. This process resulted in 61 data points per acquisition (representing the 61 DWIs) at each GM/WM surface vertex point, which were written out to separate files that could be used to visually check the diffusion data on the folded and unfolded surface and for export to Matlab 7.10 (MathWorks, Natick, USA) for further processing. At each surface vertex, we saved the unique vertex ID, the x, y, z coordinates of the vertex, the x, y, z components of the local normal vector (**n**), a unique voxel ID (i.e. because the anatomical images had a higher spatial resolution than the DWIs multiple surface vertices may sample a single, coarser DWI voxel), and finally, the 61 image intensity values extracted from the DWIs.

### Spherical Harmonics

A spherical harmonic model was fit to the log HARDI data to obtain the apparent diffusion coefficient profile *f* as in [33]. This model includes spherical harmonic terms up to the 6^th^ order from which seven types of orientationally invariant features of the HARDI profile were computed. Features 1, 2 and 7 are independent of the local normal and fully orientationally invariant features, 3–6 are relative to the local normal, **n**. Specifically,

1. The mean of the ADC profile
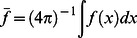
(1)
2. The k^th^ moment of *f* for k = 2.10
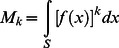
(2)where S is the unit sphere.
3. The value of *f* along **n** to the local cortical surface
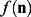
(3)
4. The mean of *f* perpendicular to the local **n**, (i.e. the mean ADC in the plane of the cortex)
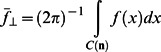
(4)where C(n) is the unit circle perpendicular to n.
5. The k^th^ moment of *f* perpendicular to **n**, for k = 2…10
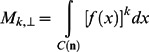
(5)
6. The two eigenvalues of the Hessian matrix of *f* evaluated at **n**. The Hessian matrix is the second derivative of the ADC profile, which expresses its curvature and is sensitive to the dispersion of fibre orientations within the tissue [31].
7. Simple rotationally invariant combinations of the spherical harmonic parameters for *k* = 0, 2, 4, 6
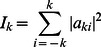
(6)where *a_ki_* is the coefficient of the spherical harmonic order *k* and index i in the series.

In total this provides a feature vector of 27 values for every vertex point, which were used to differentiate distinct cortical areas. A principal component analysis suggested that the data actually contained around 9 or 10 significant degrees of freedom. However, to test the discriminative potential of the features, the full feature vectors were used as input to an off–the–shelf support–vector machine (SVM) classifier (http://www.csie.ntu.edu.tw/~cjlin/libsvm/).

**Figure 1:**
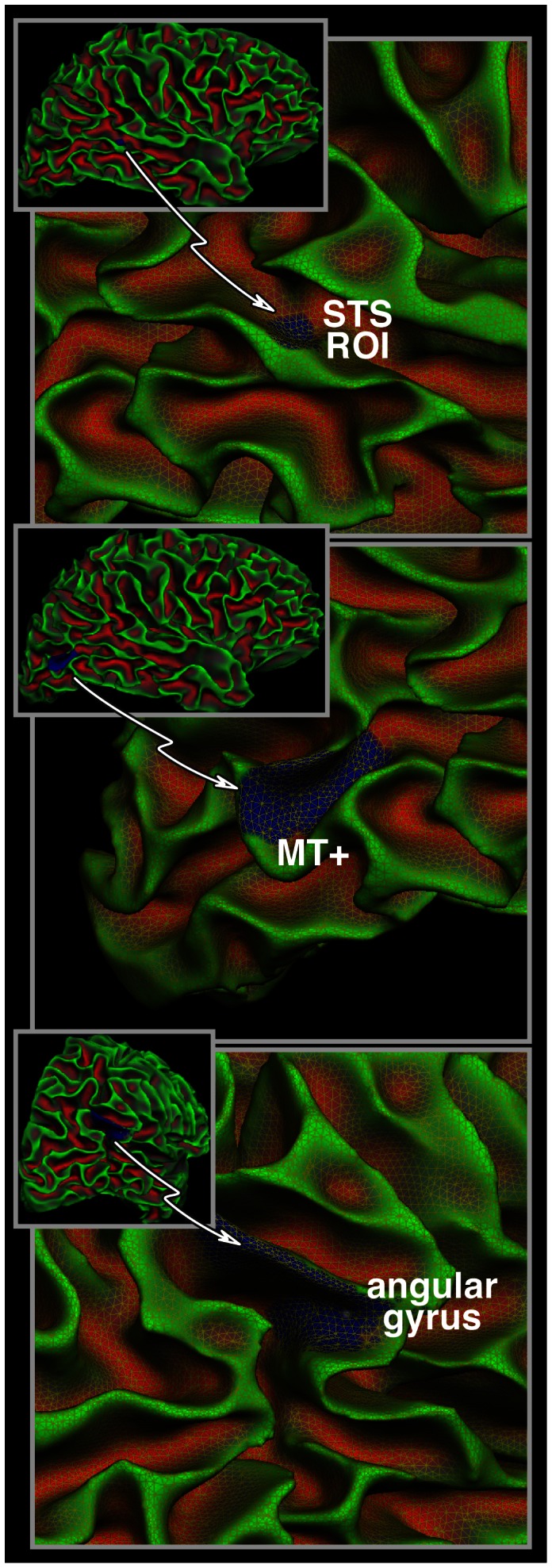
Depiction of selected ROIs on the convoluted cortical surface. Due to the convoluted nature of the cortical sheet even small, functionally and histologically homogeneous regions will have varied spatial orientation. Three of the areas investigated in this study are depicted. The background color indicated local cortical curvature (not gyrification). The mesh edges connect surface vertices.

In Experiment 1, data from the first acquisition were used to train the three–way SVM classifier on the full set of feature vectors from every vertex point within three regions. In Subject 1 (male) these three regions were MT+ (Extended middle temporal area based on retinotopy and quantitative T1 data [48]), Ang (a nearby region of the angular gyrus within the so–called “default mode network” that is known to be lightly myelinated [49]) and STS ROI (a visually responsive part of the superior temporal sulcus), as displayed in Figure 1. An additional region, just anterior to MT+, was not included in training the classifier on data from the 1^st^ acquisition but data from that region from the 2^nd^ repetition was subsequently classified to investigate whether the classifier would find borders of regions automatically. In Subjects 2 and 3 (1 male/1 female) three regions were defined solely on quantitative T1 data [48]. The regions used for the 3–way classification were Ang (as above for Subject 1), the region anterior to MT+ and M–I (primary motor cortex).

The classification rate of the classifier on the second, unseen, acquisition provides an indication of discriminability of different cortical areas based on the HARDI signal. The classification results were also painted on the cortical surface using FreeSurfer for visual representation.

In order to test the method on a larger number of distinct regions, in Experiment 2 data were extracted from additional areas. For Subject 1, nine areas were chosen as in [48] using a combination of independent anatomical and functional criteria: quantitative T1 for primary sensory/motor areas and retinotopic functional imaging data for the remaining areas. Namely, A–I,R = primary auditory cortex and rostral area; FST = fundus of the superior temporal sulcus area; IPS1 = Lateral intraparietal sulcus area 1; IPS2,3 = lateral intraparietal sulcus areas 2 & 3; S–I = primary somatosensory cortex (areas 3b,1,2); V1 = primary visual cortex; V3A = V3 Accessory; V6 = visual area 6 and VIP = ventral intraparietal area. For subjects 2 and 3, seven regions were defined in addition to the Ang, region anterior to MT+ and M–I solely on quantitative T1 data. These were MT+, A–I,R, S–I, V1, V3A, V6 and VIP. The MT+ and the region anterior to it were included to specifically test the discriminability of adjacent regions. The ability of the SVM to distinguish the regions was tested pair–wise on data from these twelve regions in Subject 1 or ten regions for Subjects 2,3. For each pair of regions the SVM was trained on data from repetition 1 and then data from the same two regions in repetition 2 were classified. Each pair–wise classification produces two results: the fraction of correctly classified voxels in each of the two regions. We average these two classification rates to get an overall classification rate for each pair. This avoids misleading high scores from unbalanced pairs of regions with very different sizes.

## Results

### Test/Re–test Analysis

**Figure 2:**
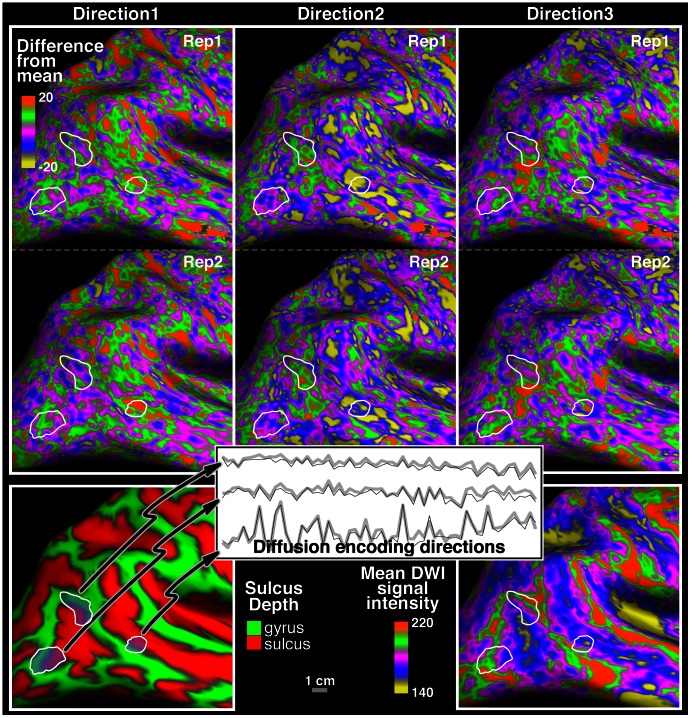
Test/re–test DWI signal intensity. The top two rows display the raw diffusion weighted image (DWI) signal intensity after the mean signal of all the 61 DWIs (see bottom right) has been subtracted from each. Three diffusion–encoding directions of both repetitions (top = Rep1, middle = Rep2) are shown. The white outlines indicate the ROIs from Figure 1. The bottom right displays the mean of the 61 DWIs. The color bar represents MRI image pixel intensity from yellow = low to orange = high. Note the different ranges between the individual images and the mean. On the bottom left gyrification of the cortex is indicated (red = sulci, green = gyri). The inset signal time–courses in the centre of the figure depict how the pixel intensity varies over the entire experiment of 61 diffusion–encoding directions within the 3 ROIs (Rep 1 = thick grey line, Rep 2 = thin black line). The scale for all images is 1 cm.

The test/re–test reproducibility was high for all 3 subjects. Figure 2 displays data from Subject 1, demonstrating that the signal variability in DWIs with varying diffusion–encoding directions is reproducible. The top 6 sub–plots display the mean adjusted raw DWI signal intensity from the posterior aspect of the right hemisphere. Data are shown for three different diffusion–encoding directions for both acquisitions. Because proximity to the coil affects pixel brightness the mean intensity of the 61 DWIs (see bottom right of figure) was subtracted from each of these six individual images. The color bar nominally represents the arbitrary MR image pixel intensity, increasing from yellow to orange. Note that while the signal intensity of a given spatial location changes as the diffusion–encoding direction is varied (from left to right in Figure 2), there is high correspondence on test/re–test (top and middle rows respectively in Figure 2). The white outlines indicate the three ROIs indicated in Figure 1 from where data was sampled before mean correction. The per–direction mean of the data for all vertices in each ROI are displayed in the inset at the centre of the figure demonstrating how the DWI signal varies as a function of 61 diffusion–encoding directions (Rep 1 = thick grey line, Rep 2 = thin black line). These ‘time courses’ are unique for each region and indicate reproducibility on test/re–test. The three plots were vertically displaced from each other only for clarity but scaled identically. Proper treatment of such data must take tissue orientation into account. Note also that even within these small regions the signal intensity can vary greatly, which is a combined effect of microstructure and spatial orientation of the tissue. For comparison, gyrification of the cortex is indicated on the bottom left (red = sulci, green = gyri).

### Classification

**Figure 3:**
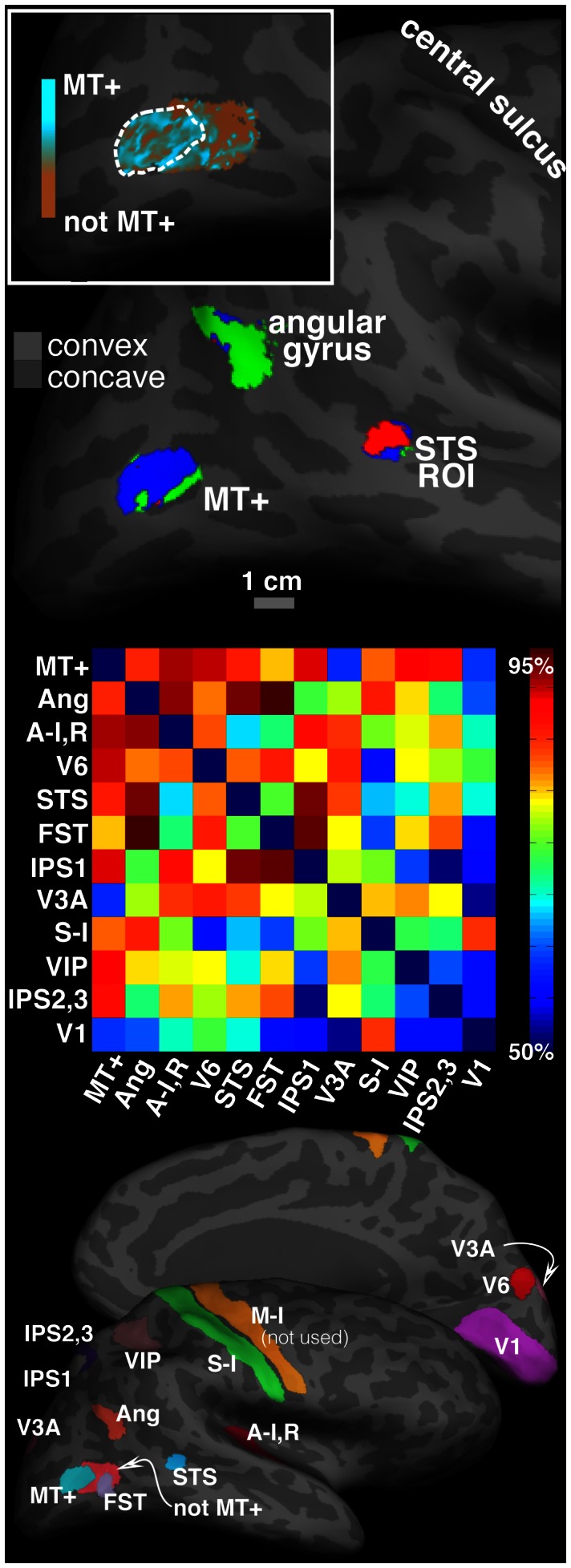
Classification results of the test/re–test data. (Top) Classification results displayed on the map of cortical curvature. After being trained on data from ROIs of the 1^st^ acquisition data from the same ROIs of the 2^nd^ acquisition are classified as MT+ (blue), Ang (green) and STS ROI (red). The reliability of the classification process is supported by the fact that data from each cortical area is classified correctly in a large connected set of vertices and only the edges are classified erroneously as one of the other tissue types. (Inset) When the area just anterior to MT+, which was unseen while training the classifier is, is included in the classification an approximate border can be identified where success of the classifier drops sharply. (Middle) The ability of the support vector machine (SVM) to distinguish pairwise data from 12 distinct cortical regions. The color code indicates percent of correct classification of data in repeat 2 after the SVM was trained on data from repeat 1. Most regions are classifiable above chance though the SVM struggles with classifying V1 (primary visual cortex) correctly. Another internal check shows that the IPS1 and IPS2,3 (IPS = lateral intraparietal sulcus), which are neighboring, functionally related parietal visual areas, are hard to distinguish. For the definitions of the abbreviations please see Methods. (Bottom) Depiction of all regions where data were extracted from one or all three of the subjects. The short descriptive names are defined in the text. M-I was used only for Subjects 2 and 3, hence it is designated (not used) to indicate that it is not included in the 12×12 matrix of Subject 1 (middle).

After being trained on data from the 1^st^ of two acquisitions, the SVM classifier attained 80–82% correct classification of the data from the three different cortical areas in the 2^nd^ acquisition for the 3 subjects. Figure 3 (top) displays the results from Subject 1, where the colors represent the class assigned to each voxel by the SVM: red, green and blue indicating that a feature vector was most like the STS ROI, the Ang or the MT+ respectively.

The results of testing the SVM on pair–wise data from the ten or twelve regions produced similar results across the 3 subjects. In general V1 was hard to distinguish. On the other hand the Ang and A–I,R were highly discriminable from the other regions in all three subjects. When looking at pairs of regions specifically, we found that V6 vs V3A, MT+ vs S–I and MT+ vs VIP were easily distinguishable in all three subjects and S–I vs V3A also achieved 70% mean classification. All of these regions are heavily myelinated. In addition, in Subjects 2 and 3, where M–1 was included as one of the regions, it was easily distinguishable from V1 and S1. In Subject 1 functionally related IPS1, IPS2,3 and VIP were hard to tell apart, which may reflect similar structure in these functionally related areas. Figure 3 (middle) displays the 12×12 results of the Subject 1.

In Subjects 2 and 3 where MT+ and the area just anterior to it were included in the pair–wise analysis, these two areas were discriminable (above 75% mean classification rate). In Subject 1 we used the MT+, Ang and STS ROI to train the classifier on data from repeat 1 and then in repeat 2 we exposed the classifier for the first time to the region anterior to MT+ to test whether a border could be found between these two regions. The inset in the top of Figure 3 shows the results, which indicate that the classifier indeed could approximately detect the edge of MT+ (although it is important to note that this result hinges on the data used from the other two ROIs). The algorithm’s ability to distinguish adjacent regions is important, because it suggests that the classifier is not sensitive only to some low frequency artifact of the imaging process allowing it to distinguish distant regions, but to differences in intrinsic tissue properties.

## Discussion

We have shown that the HARDI signal in cortical GM is dependent on the diffusion–encoding direction in a highly reproducible manner. This dependence may be taken to be a fingerprint of the underlying tissue, which is sensitive to the microstructural differences that exist between different cortical regions. Non–invasive MR–based parcellation of the cortical mantle has recently attracted considerable interest [19], [25], [38], [48], [50], [51]. The diffusion–based method introduced here combines off–the–shelf tools and surface–based analysis to demonstrate that gray matter diffusion patterns can be used to distinguish cortical areas. Additional opportunities for future development are outlined below.

The DWI signal in a given GM voxel is not isotropic but changes subtly for different diffusion–encoding directions. Our results indicate that this variability is reproducible on test/re–test. Although we must be careful to exclude systematic error, we think this variation arises from the interaction of the underlying tissue microstructure with the diffusion–encoding direction. There is previous evidence that GM microstructure is not simply isotropic. For example, histological stains show myelinated axons within the cortex in both radial as well as tangential directions [26], [27]. Some areas are known to be anisotropic in a tangential direction (e.g. V2 cytochrome oxidase stripes) [52]. If different cortical areas had varying proportions of myelinated fibres in these two populations, it would likely result in corresponding changes in the direction spectrum of the DWI signal intensity. Moderately high diffusion anisotropy in cortical areas has been reported at 7T [25] and a few other recent reports used DWIs to distinguish areas in the gray matter [38], [51]. In each case the underlying tissue is discriminated based on the DWI signal. Our approach offers the advantage that it uses a non–parametric model of the signal via spherical harmonics, which provides a much richer set of features for fingerprinting the underlying tissue. By contrast, McNab et al. [51] concentrated on the principal orientation of the diffusion tensor relative to the local cortex normal vector – a two dimensional feature, drawn from a very rich data set acquired with 256 diffusion encoding gradient directions. Haroon et al. [38] use more dimensions in their method, by measuring the number of peaks in the Q–ball ODF after bootstrap iterations – but this method still typically only uses a feature vector with four dimensions. The feature vector we use here has 27 elements. Although there is significant redundancy between those elements (PCA suggests the vectors have intrinsic dimension of 9 or 10), it provides a much richer feature set allowing more reliable and finer discriminability. Many of our features are directly referenced to the local orientation of the cortex. The feature vector also extends naturally to include more elements should they prove useful with the data acquired or for the question investigated. For example features 2 and 5 could include even higher order moments and feature 7 could include additional elements if the spherical harmonic series was truncated at a higher order. Here we intentionally included more orders than we believe useful to make sure we had as complete a description of the data as possible.

The set of features also includes rotationally invariant features of the diffusion signal profile. The problem of identifying a minimal set of independent features that includes all rotationally invariant aspects of the profile is a topic of on–going research for which the current literature contains no solution. We choose a large redundant set to capture as much information as possible. A more compact set may be identified, to improve the computational efficiency without compromising classification/segmentation performance. We note recent work on complete sets of orientationally invariant features of spherical functions (e.g. [53]), which may be able to compact the same information into smaller sets of features.

### SVM Classification

We emphasize that the classifier used no regional spatial information (aside from the feature reliance on the local surface normal), but only voxel–wise feature vectors. This suggests the data contain information on which to base a cortical parcellation. When the classifier was trained with data from 3 distinct clusters of the 1^st^ acquisition the classification of the 2^nd^ dataset resulted in largely connected sets, which correctly identified the actual cortical area (Figure 3 Top), even though the algorithm did not require this contiguity. In this Subject the classification results were erroneous only near the edges of each of the ROIs. This however, may not be a failure of the method. It could indicate that the ROI selected actually included tissue that was truly histologically different or reflect partial volume effects due to the lower (2.3 mm) DWI resolution. The inset at the top of Figure 3 shows that the area anterior to the MT+ region seems to be distinct as identified by the natural border beyond which the classification results drop.

When the classifier was used in a similar fashion on pairwise comparisons of the data from the 10 or 12 distinct cortical regions not every area could be distinguished from every other one (Figure 3 middle). The ability of the classifier to tell the two regions apart could be taken as a measure of similarity (i.e. discriminability) of the underlying tissue. For example for Subject 1 the data from IPS1, VIP and IPS 2,3 in the 2^nd^ acquisition are difficult to tell apart after data from the same regions in the 1^st^ acquisition was used to train the classifier. This result supports the idea that cortical tissue is similar in these functionally related areas, which are often co–activated in neuroimaging studies [54]. Another example is MT+, which the classifier easily distinguishes from most other regions in all three subjects. Based on the fact that, histologically, MT+ is distinct in having particularly dense myelination as well as very high levels of cytochrome oxidase it may be expected that it is easily identifiable. Note that the current implementation takes a single measure of the GM signal in the DWIs midway through the cortex, which is unlikely to capture a full histological specialization of the local GM (see below). The classifier may perform better on higher resolution DWI data: at higher resolution several samples could be taken from different depths to increase the size of the feature vector or a single cortical layer could be more specifically targeted.

**Figure 4:**
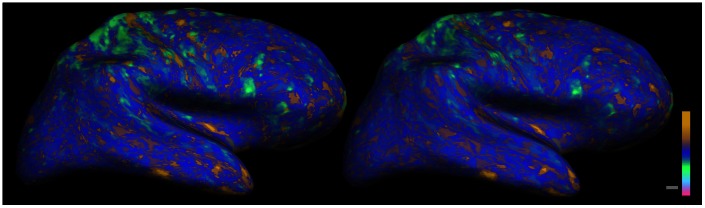
Unsupervised clustering using k–means. The feature vectors for all vertices in a single hemisphere were fed to a k–means clustering algorithm to produce 40 distinct clusters. The color bar runs from 1 (red) to 40 (green) where clusters with similar feature vectors are colored similarly. Clusters were sorted in such a way as to minimize the sum of Euclidean distances between consecutive cluster centres. The reproducibility of results between the two repeats is striking. Data from Subject 1 are displayed.

Our principal aim with this work was to demonstrate and establish that the voxel–wise HARDI signal is reliably reproducible and is discriminative of distinct GM areas. In a practical situation, rather than a test/re–test scenario, the aim would be to directly parcellate the cortex in an unsupervised manner, based on a single acquisition or to classify an individual’s data using supervised training on a larger cohort. As an example of unsupervised classification, simple k–means clustering produces reproducible results on a single data set without the need for a training data set (Figure 4). It seems likely that including some spatial information in the feature vector to allow spatially separated regions with similar HARDI signal to form separate clusters would further improve the performance. A maximally effective parcellation technique based on MRI would probably need to include complementary information in the form of (population–based) architectonic maps as well as data with different MRI contrast(s) from the same individual.

Significant further improvements could come from a richer acquisition protocol including multiple HARDI shells with different b–values, as in, for example in [55] or [35], as the different shells generate sensitivity to different features of tissue microstructure. With our b–value of 1000 s/mm^2^, the dominant tissue feature likely to contribute to signal variation is the orientation distribution of neurites [56], [57]. Including additional shells at higher b–values and varying diffusion times could provide sensitivity to other features, such as fibre size and density [36].

### Limitations and Future Directions

One critical issue is precisely and accurately detecting the GM/WM and pial surface boundaries in image–based methods. This is not a limitation of the software used. Rather, the reduction of myelin is gradual as the WM fibers enter the deeper layers of the cortex and hence the GM/WM boundary is difficult to define. The pial surface can also be difficult to locate where it approaches itself closely. We attempted to minimize the effect of GM/WM boundary detection by sampling the DWI signal halfway between these two surfaces along the local normal. A related limitation is that the layers vary in thickness among the cortical areas. Therefore, even if the GM/WM boundary detection was perfect, a fixed fraction along the local normal may sample different cortical layers at different vertex points. The resolution (2.3 mm) and b–value (1000 s/mm^2^) used here were chosen to ensure high signal level and to minimize eddy current and susceptibility induced distortions. Improving the resolution and diffusion–encoding strength is recommended but it is important to maintain good image quality. For example, due to susceptibility–induced distortions of the EPI images, parts of the frontal and temporal lobes are distorted in single–shot EPI. As a result the exact co–registration of the T1–weighted anatomical and HARDI data was difficult even at 2.3 mm resolution. More advanced acquisition and correction methods [58]–[61] could help reduce these distortions. Also, at the 2.3 mm isotropic resolution it is likely that partial volume effects can occur between the grey and white matter leading to classification that is not solely dependent on the grey matter signal. While this, in principle, is not a limitation, the interpretation is different because the cortical areas are not discriminated on signal from the grey matter alone. We mention here that a different and larger set of regions were used in the SVM classification for Subject 1 simply because more fMRI data was available for that subject to define ROIs. However, we do not consider this a limitation because the method presented here is expected to work irrespective of the cortical area from which the data was extracted.

We demonstrated that the minimal condition of reproducibility is satisfied. This is a necessary but not sufficient condition for reliable cortical parcellation. Future extensions of this study are required for full validation. Consistency across subjects was demonstrated here but need to be established on larger cohorts. Next, cross–validation of parcellation results with other imaging and histological modalities will help establishing its construct validity. The relevance of the DWI based parcellation can also be further evaluated by correlation with behavioral and other individual measures such as ageing.
